# Neck Abscess as a Rare Sequela of Pediatric Varicella-Zoster Infection: A Case Report

**DOI:** 10.7759/cureus.77639

**Published:** 2025-01-18

**Authors:** Deivanraj Palanimuthu, Enci Yong, Komathi Ramachandran, Irise Hoi Khin Chen

**Affiliations:** 1 Otorhinolaryngology - Head and Neck Surgery, Hospital Putrajaya, Putrajaya, MYS; 2 Radiology, Hospital Putrajaya, Putrajaya, MYS; 3 Otorhinolaryngology - Head and Neck Surgery, Hospital Kuala Lumpur, Kuala Lumpur, MYS

**Keywords:** cervical neck abscess, head and neck abscess, pediatric deep neck space infections, varicella-zoster, varicella zoster (chicken pox)

## Abstract

Chickenpox is caused by the varicella-zoster virus (VZV), a DNA virus of the herpes virus family. While typically mild and self-limiting, it poses a high risk of contagion. A generalized pruritic rash is the common initial symptom among pediatric populations. Complications often include bacterial infections of skin lesions, predominantly staphylococcal or streptococcal, which may progress to cellulitis or bullous impetigo. Additionally, it may lead to more severe complications such as cerebellar ataxia, encephalitis, viral pneumonia, hemorrhagic conditions, and joint involvement. We present a case of a 17-month-old male diagnosed with varicella-zoster infection a week prior. His presentation included diffuse swelling in the left neck, accompanied by fever and reduced oral intake. Clinical and radiological assessments confirmed a left neck abscess with multiple cervical lymphadenitis. Following the incision and drainage of the abscess, the patient achieved complete resolution of symptoms with antimicrobial treatment. To the best of our knowledge, this is the first reported case of pediatric varicella-zoster infection complicated by a secondary bacterial skin infection leading to a neck abscess. It underscores the importance for clinicians to perform comprehensive evaluations in patients manifesting secondary skin lesion infections. Early referral to tertiary care facilities is imperative, as neck abscesses, albeit rare, necessitate prompt treatment to avert potential complications.

## Introduction

Chickenpox, also known as varicella, is an extremely contagious infection caused by the varicella-zoster virus (VZV). It typically presents as a primary infection in individuals with reduced immunity and is characterized by a vesicular skin rash that evolves into scabs. The rash commonly begins on the chest, back, and face before spreading to other parts of the body. In some individuals, VZV may remain latent in the sensory ganglia and get reactivated later in life, resulting in herpes zoster [1]. The associated prodromal symptoms in adolescents and adults often include fever, fatigue, myalgia, pharyngitis, and headaches, typically persisting for approximately a week. In pediatric populations, the illness may not be preceded by prodromal symptoms, and the initial sign could be a rash or oral cavity lesions [2].

Varicella is usually self-limiting, resolving within two to four weeks; however, in some cases, it can lead to severe complications, such as pneumonia, encephalitis, bacterial skin infections, and arthritis, with secondary bacterial infections being particularly common [3]. These infections are often caused by Streptococcus pyogenes or Staphylococcus aureus, with cellulitis, impetigo, and erysipelas being frequent manifestations [4]. A rare but serious sequela of varicella infection is the development of deep neck infections, such as Ludwig's angina or neck abscesses. Surgical drainage of neck abscesses and appropriate antimicrobial therapy are the cornerstones of neck abscess management [5]. To date, only one such case has been reported, involving a retropharyngeal abscess, caused by a sequela of tonsillitis that led to the spread of infection to the potential space [6]. This case represents the first instance of pediatric varicella-zoster infection complicated by a secondary bacterial skin infection and highlights neck abscess as a potential complication.

## Case presentation

**Figure 1:**
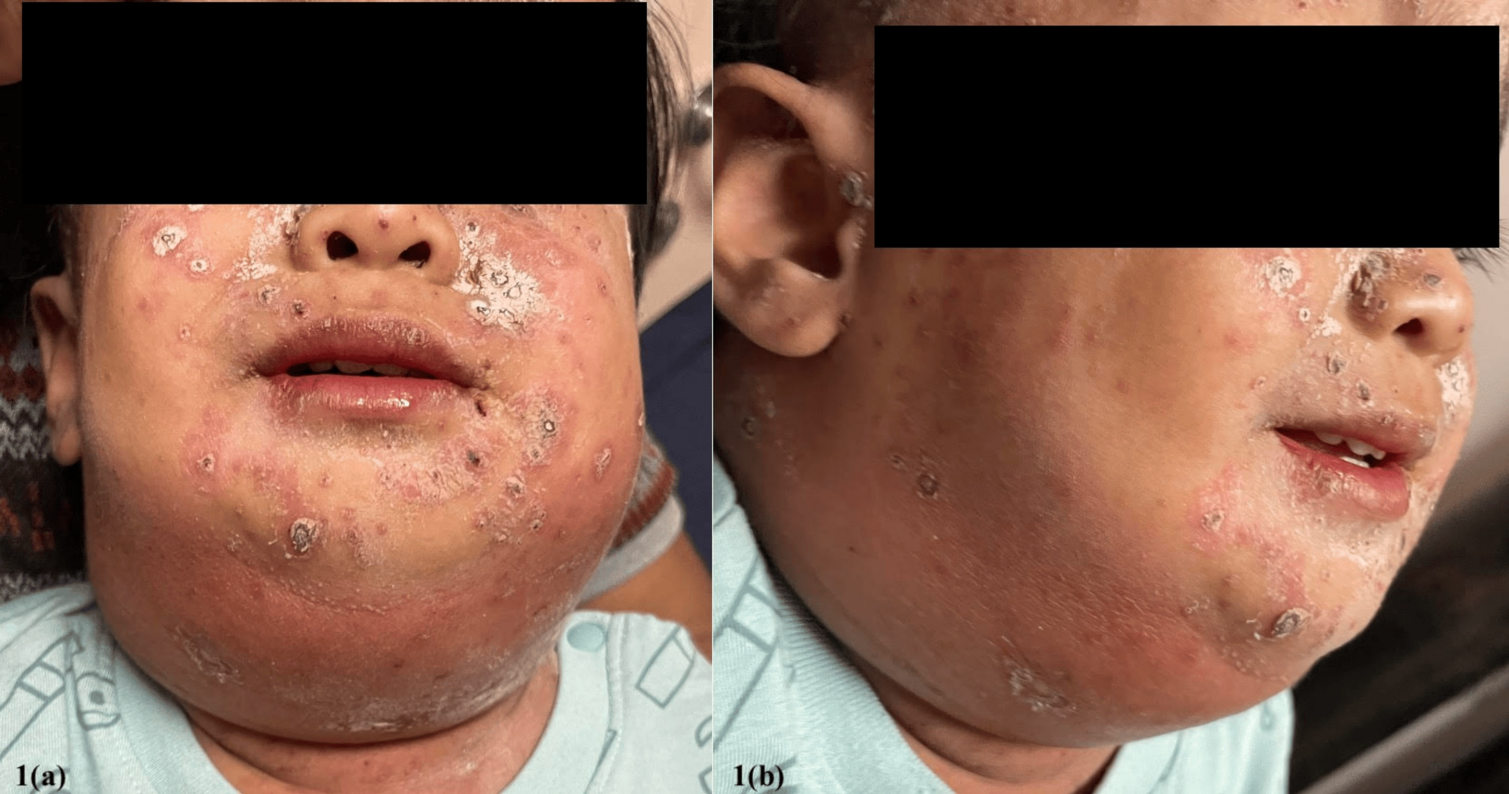
Clinical picture of neck examination (a, b) Diffuse swelling extending from the left pre-auricular region to the infra-auricular, submandibular, submental, and contralateral areas, accompanied by erythematous skin and dry crusting

A 17-month-old male presented to the emergency department with progressively worsening bilateral painful neck swelling for three days, accompanied by a documented fever of 39 °C and poor oral intake. The initial neck swelling was localized to the left pre-auricular region, later extending to the left submandibular and submental areas. No obstructive symptoms such as noisy breathing or dysphagia were reported. The neck swelling was aggravated by the skin pustules over the left pre-auricular region, where he had been diagnosed with varicella-zoster skin infection one week before presentation. Upon general examination, the child appeared septic but was not in respiratory distress. Dry crusted lesions were observed all over the body, with no wet or weeping lesions. Examination of the neck revealed diffuse swelling extending from the left pre-auricular region to the infra-auricular, submandibular, submental, and contralateral neck, with erythematous skin and dry crusting (Figures 1a, 1b).

Multiple shotty cervical lymph nodes were palpable, with the largest at the left level V measuring 1 x 1 cm, mobile, and non-tender. Oropharynx examination revealed multiple vesicle lesions over the mucosa and inflamed left Wharton’s duct without raising the floor of the mouth. Other subsites of the oral cavity and oropharynx appeared normal.

**Figure 2:**
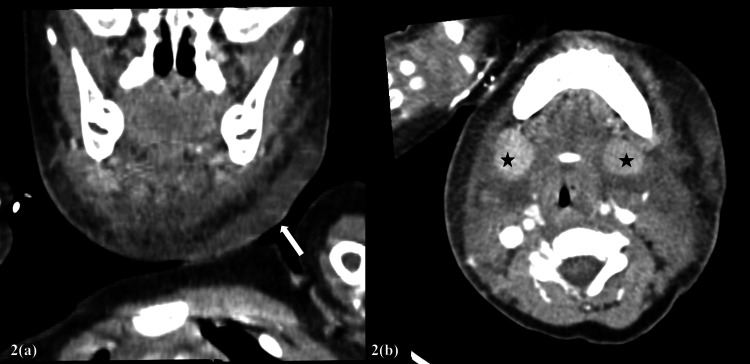
CECT of the neck (a) Features of Ludwig's angina (white arrow), with no evidence of an abscess; and (b) bilateral submandibular sialadenitis (black star) CECT: contrast-enhanced computed tomography

Given the presenting history and clinical features, a diagnosis of varicella-zoster infection complicated with neck cellulitis was made. Flexible nasopharyngolaryngoscopy (FNPLS) revealed normal findings. Laboratory tests showed a raised total white cell count of 33 x 10^9^/L with neutrophil predominance and elevated C-reactive protein. Ultrasonography of the neck revealed cellulitis changes at the anterior upper neck, bilateral parotitis, and reactive lymphadenopathy. Contrast-enhanced CT (CECT) of the neck showed findings suggestive of bilateral parotid and submandibular sialadenitis complicated with Ludwig angina causing airway narrowing, with no evidence of abscess at the time of the study (Figure 2). 

Despite 72 hours of optimal antimicrobial treatment with intravenous ceftriaxone (dose), the neck swelling increased in size with an area of localized fluctuancy. Incision and drainage were performed under general anesthesia, draining 30 cc of frank pus. The culture and sensitivity revealed Streptococcus pyogenes sensitive to clindamycin and penicillin G. Daily wound dressing was performed, allowing the wound to heal by secondary intention. At a two-month follow-up in the outpatient clinic, the neck wound had completely healed, and the patient was well.

## Discussion

This report highlights a rare but serious complication of varicella: the development of a neck abscess secondary to bacterial superinfection. Secondary bacterial infections in varicella are commonly caused by Streptococcus pyogenes and Staphylococcus aureus, especially in cases with severe or extensive skin involvement where lesions become infected. The progression to Ludwig’s angina and later neck abscess, as observed in this patient, is rare but can result in significant morbidity and mortality due to the potential for airway compromise. This underscores the importance of early recognition and prompt intervention in pediatric patients presenting with rapidly progressive neck swelling and other signs of deep neck infections during a varicella illness.

The treatment of secondary bacterial infections in varicella involves the administration of appropriate antimicrobial therapy, often tailored based on culture and sensitivity results, and surgical drainage if abscess formation occurs. Commonly used antibiotics for treating infections caused by Streptococcus pyogenes include penicillin G and clindamycin, which are effective against this pathogen [7]. Our patient was successfully managed with intravenous ceftriaxone, followed by culture-directed therapy, leading to favorable outcomes.

While varicella is generally a mild disease, the risk of severe complications, including bacterial superinfections, is more significant in certain populations, including adults and those with immune suppression. The incidence of secondary bacterial infections in varicella is variable but has been reported in up to 30% of cases, particularly those with severe or extensive skin involvement [8]. Our review of the literature revealed four reported cases of spinal epidural abscess as complications of varicella, with group A beta-hemolytic Streptococcus and Staphylococcus aureus being the most commonly identified pathogens [9-11]. Other reported cases of varicella-related abscesses include infections in the extremities, back, and mediastinum, caused by similar pathogens [12-14].

Notably, deep neck infections complicating varicella are rare. Among these, only one case has been reported to involve a retropharyngeal abscess, which was linked to tonsillitis and subsequent infection spread [6]. The current report contributes to the literature by documenting an uncommon neck abscess resulting from a secondary bacterial infection following a varicella-zoster infection in a pediatric patient.

## Conclusions

This report highlights the significant risk of severe complications that can arise from VZV infection, with a particular focus on the development of secondary neck abscesses. While such complications are rare, they can have life-threatening consequences if not promptly identified and managed. Timely recognition of the condition is essential, as the initiation of appropriate antimicrobial therapy and prompt drainage of any formed abscesses are critical to prevent further progression. Furthermore, this report emphasizes the importance of considering deep neck space infections in pediatric patients with varicella, especially when they present with symptoms such as progressive neck swelling and fever. These signs should raise suspicion for more serious complications beyond the typical cutaneous manifestations of varicella. Given the potentially severe outcomes of the delayed intervention, early imaging and culture-directed therapy are crucial for guiding treatment and optimizing patient outcomes.
